# Development of a novel mobile application to detect urine protein for nephrotic syndrome disease monitoring

**DOI:** 10.1186/s12911-019-0822-z

**Published:** 2019-05-30

**Authors:** Chia-shi Wang, Richard Boyd, Russell Mitchell, W. Darryl Wright, Courtney McCracken, Cam Escoffery, Rachel E. Patzer, Larry A. Greenbaum

**Affiliations:** 1 Division of Pediatric Nephrology, Emory University, 2015 Uppergate Drive NE, Atlanta, GA 30322-1015 USA; 2 Children’s Healthcare of Atlanta, Atlanta, GA USA; 3 Georgia Tech Research Institute, Georgia Institute of Technology, Atlanta, GA USA

**Keywords:** Nephrotic syndrome, Mobile applications, Urinalysis, Children

## Abstract

**Background:**

Home monitoring of urine protein is a critical component of disease management in childhood nephrotic syndrome. We describe the development of a novel mobile application, UrApp – Nephrotic Syndrome Manager, to aid disease monitoring.

**Methods:**

UrApp was iteratively developed by a panel of two pediatric nephrologists and three research engineers from May 2017 to October 2018 for Apple iPhones. App features were devised by this expert panel to support urine monitoring and other home care tasks. Each feature and user-app interface element was systematically reviewed by the panel and iteratively redesigned to remove anticipated use issues. The app prototype was then refined based on two rounds of usability testing and semi-structured user interviews with a total of 20 caregivers and adolescent patients. The analytic function of UrApp in providing a camera read of the urine test strip was compared to a standard urinalysis machine using 88 patient urine samples and three iPhones, model versions 6S and 7. Exact agreement and weighted kappa were calculated between the UrApp and urinalysis machine reads.

**Results:**

The final UrApp features include: camera read of a urine test strip; analysis of urine protein trends and alerts for new disease relapse/remission; transmission of urine protein results to providers; education materials; and medication reminders. During the second round of UrApp usability testing, all users were able to perform each of the functions without error and all perceived UrApp to be helpful and indicated that they would use UrApp. UrApp camera results had 97% exact agreement and an overall weighted kappa value of 0.91 (95% CI, 0.85–0.97) compared with standard urinalysis machine interpretation.

**Conclusions:**

UrApp was specifically designed to support patients and families living with nephrotic syndrome by supporting disease monitoring and home management tasks. The technically innovative feature that makes this possible is the use of a smartphone camera to read the urine test strip. This novel tool has the potential to improve disease monitoring and reduce management burden.

**Electronic supplementary material:**

The online version of this article (10.1186/s12911-019-0822-z) contains supplementary material, which is available to authorized users.

## Background

Idiopathic nephrotic syndrome is one of the most common chronic kidney diseases in children and has a relapsing and remitting course in approximately 80% of the patients [1, 2]. Home urine testing for protein is recommended to detect disease relapse and remission in a timely manner. Cargivers are instructed to use urine reagent test strips - thin plastic strips with small blocks of paper impregnated with various regeants that produce visible colorimetric reactions once in contact with urine. One reagent block specifically produces a semi-quantitative result for urine protein that can be read by eye [3]. New proteinuria signals disease relapse before the development of overt symptoms such as edema. Thus, caregivers are to alert their providers to the occurrence of proteinuria in a timely manner so that treatment can be initiated or adjusted to treat each relapse and prevent disease complications. It is also important for caregivers to track urine protein for resolution so that treatments, generally corticosteroids, can be tapered or stopped to minimize side effects. Disease relapse is defined as urine protein ≥2+ (100 mg/dL) for 3 consecutive days and remission is defined as negative/trace urine protein for 3 consecutive days [2].

These monitoring demands are taxing in that families must remember to check the child’s urine, recall results, understand what the results mean, and follow complex medication regimens that are changed often and have significant side effects. Focus group and individual caregiver interviews with parents caring for children with nephrotic syndrome have found that parents report difficulty understanding the significance of urine protein results and knowing when to report and act on significant changes [4].Unfortunately, approximately half of patients are nonadherent with urine protein monitoring and medications in a single-center study [5].

Mobile health applications (mHealth apps) is a rapidly growing field in disease management. Currently, 77% of U.S. adults own smartphones, and ownership is highest among younger adults (18–29 year-olds: 94%; 30–49 year-olds: 89%) [6]. The ubiquitous presence of smartphones with advanced computing and communication capabilities, large memory, high quality cameras and other sensing capabilities are used to support home care for patients with chronic medical conditions and improve access to information [7, 8]. Self-management support and clinical information systems are identified by the Chronic Care Model (MacColl Institute for Healthcare Innovation at Group Health Cooperative) as two key elements in effective care of chronic conditions. Viewed through this framework, mHealth apps can be effective interventions for chronic disease management [9]. In asthma, mHealth apps have been used to track day-to-day symptoms and provide individualized, timely reminders and education leading to improved clinical outcomes (e.g., symptoms, lung function) and patient-reported outcomes (e.g., adherence, self-efficacy, quality of life) [10]. Similarly, mHealth apps for diabetes self-management have used smartphones to track home health data, including blood glucose, diet, and activity level. Data are used to provide personalized feedback such as insulin dosages and lifestyle coaching. While there are mixed results, a number of studies showed that app use resulted in improved HbA1c levels [11].

In nephrotic syndrome, there are numerous aspects of self-management that may be facilitated by a mobile app. First, the visual analysis of a urine test strip is subject to human error, including reading the wrong reagent block and erroneous assessments of color. This can be improved through using a smartphone’s camera and CPU to read and analyze test strip results. Apps, with their inherent interactivity, can provide reminders for urine testing, capture the results, and analyze trends to detect disease relapse/remission. Apps can also alert a caregiver to seek medical attention and directly transmit test results to providers. Lastly, apps can provide medication reminders for nephrotic syndrome patients who are on highly complex medication regimens. These mHealth capabilities support numerous components of effective management detailed by the Chronic Care Model: accurate assessment, action-planning, follow-up in self-management support, timely reminders for providers and patients, clinically relevant data tracking, and information sharing between providers and patients in clinical information system design [12].

To support patients and families in self-managment and to improve disease monitoring, we built UrApp - Nephrotic Syndrome Manager, a mobile application (app) which uses the Apple iPhone camera to interpret urine test strip protein results. UrApp also tracks and analyzes urine protein trends, alerts users and providers of significant findings, and provides patient education and reminders. This paper describes the formative development of UrApp beginning with theory-informed design of app features; technical development and analytic validation; and iterative refinement of app features based on usability testing and user-input.

## Methods

### Overview of UrApp development

**Fig. 1:**
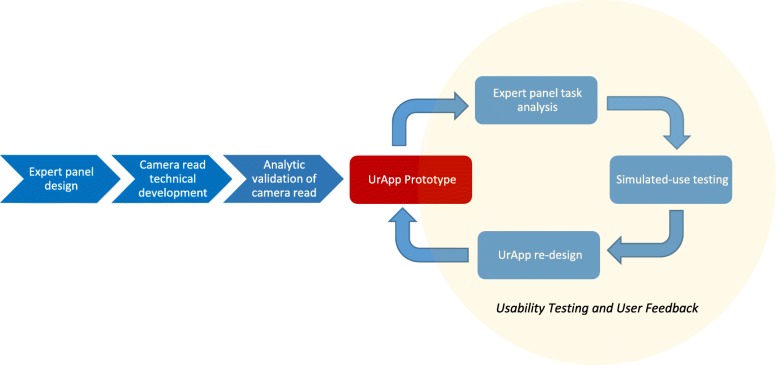
Development process of UrApp

In this prototype development research, UrApp was developed from concept to prototype through: 1. theory-informed conceptual design of app features by content experts, 2. technical development including the innovative feature of camera read of a urine test strip, 3. analytic validation of the UrApp camera read compared to a standard urinalysis machine, and 4. iterative refinement of UrApp features based on usability testing and user feedback (Fig. 1). UrApp development took place from May 2017 to August 2018.

### Theory-informed conceptual design

Two pediatric nephrologists (CW and LG) and three engineers with expertise in image processing, health app development, and human-mobile interaction (RB, RM, DW) formed the UrApp expert panel. The panel brainstormed and met to conceptualize UrApp specifications to support components of effective management according to the Chronic Care Model [12] specific to childhood nephrotic syndrome management. Throughout this conceptualization phase, mock-ups of the app were made, reviewed, and revised until the panel agreed with the specifications. Table 1 details the six functions designed by the expert panel, the nephrotic syndrome tasks they support, and relevance to chronic care management per the Chronic Care Model.

**Table 1:** Expert-panel conceptualized UrApp functions and the nephrotic syndrome tasks and Chronic Care Model elements they support

Function	Nephrotic Syndrome Management Task	Chronic Care Model Element
a) Urine test strip camera read	Automatic reading of urine protein square on test strip to support visual interpretation of the test result	**• Self-management support**: provides basic information; facilitates disease assessment; emphasizes patient’s central role in care
b) Alerts for significant urine protein findings	Automatic tracking and analysis of urine protein trends to support: results recall, analysis of clinically meaningful changes in results (relapse, remission), and decision-making on when to contact physicians and how often to perform urine testing	**• Self-management support**: provides basic information; facilitates disease assessment; emphasizes patient’s central role in care
		**• Clinical information systems**: provide timely reminders for patients; summarizes data; shares information with patients
c) Transmission of urine protein results	Facilitates communication with providers to reduce burden and provide results documentation	**• Clinical information systems**: identifies subpopulations for proactive care; facilitates individual patient care planning; shares information with providers to coordinate care
		**• Delivery system design**: plans interactions and follow-up individualized to disease severity
d) Urine protein documentation with graphic display	Summary displays of urine protein test results with color-coding in both graphic and calendar format promotes understanding of urine protein trends and supports result documentation	**• Self-management support**: provides basic information; facilitates disease assessment
		**• Clinical information systems**: summarizes data, shares information with patients
e) Education materials	Documents and videos on relevant disease management information helps families understand disease and treatment complications and how to monitor for them, follow disease-specific diets, how to perform urine testing	**• Self-management support**: provides basic information; emphasizes patient’s central role in care; organizes resources to provide ongoing support
		**• Decision support**: shares information with patients to encourage their participation
f) Medication and urine testing reminders	Reminders support adherence and accuracy with medication and urine testing.	**• Clinical information systems**: provides timely reminders for patients
		**• Self-management support**: provides strategies to increase medication and urine testing adherence

### UrApp technical development

Technical development of UrApp was carried out by the engineers on the expert panel. UrApp was developed for Apple iPhones running the iOS 11 operating system, with Roche’s Chemstrip 2GP® (Indianapolis, IN) urine dipsticks, a widely available and validated urine dipstick [13]. The app was developed in the Swift and C++ programming languages using Apple iOS Software Development Kit and OpenCV.^6^ Unit testing was performed throughout the course of UrApp development.

The image processing for urine dipstick protein interpretation contains two steps. First, UrApp calibrates the camera to the environment’s lighting conditions by capturing an image of a white sheet of paper and automatically adjusts the camera’s color temperature. Next, an unused Chemstrip 2GP® test strip is imaged to test the calibration of the camera. If the camera produces a color reading within a predetermined tolerance, the calibration is deemed successful and testing may proceed.

**Fig. 2:**
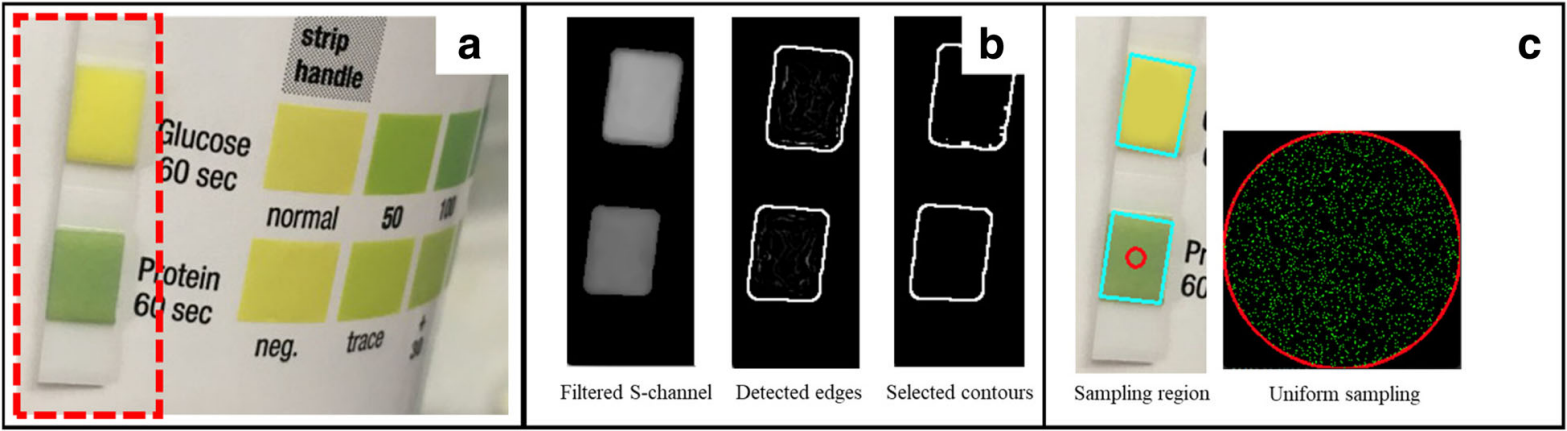
UrApp colorimetric analysis of the dipstick urine protein square: **a** extraction of the ROI, **b** edge detection of the dipstick test squares, **c** color sampling within the protein test square

In the second step, UrApp performs a colorimetric analysis of the urine protein square on Roche’s Chemstrip 2GP® test strip. UrApp uses 30 frames of video (approximately 1 sec of elapsed time) to perform the analysis. A small region of interest (ROI) is extracted from each video frame based on alignment of the test strip to guides shown on the user’s screen (Fig. 2a). The true color image is converted to the hue-saturation-value system for analysis. A Laplacian edge detector with a kernel width of five pixels is applied to the S-channel, and a noise-reduction technique is applied to select the contours of the protein square (Fig. 2b). UrApp uses the contour to sample pixels near the center of the protein square, and a uniform sampling technique is employed to select 64 equally-weighted random locations within the center (Fig. 2c). The hue values of the 64 locations (H-channel) are averaged for each of the 30 image frames. All 30 sample means are then averaged, which provides a well-defined and theoretically normally-distributed estimate of the mean hue value over the 1 sec sampling interval. Titration experiments using protein-containing solutions (water and egg white mixtures), with protein level confirmed by standard urinalysis machine reads, were performed to determine the hue values at the boundaries of the trace, + 1, + 2, and + 3 regions. These edge transition values are used to convert each hue measurement to a protein concentration result by a simple binning process.

### Image processing validation

The performance of UrApp camera interpretation of urine protein dipstick results was compared to a standard urinalysis machine (Urisys 1100, Indianapolis, IN) using three iPhones, model versions 6S (one phone) and 7 (two phones) in 88 patient urine samples. Exact agreement and weighted kappa were calculated between UrApp and standard machine reads. Exact agreement between the two iPhone models were also determined. All statistical analyses were performed using SAS Version 9.4 (Cary, NC).

### Usability assessment and user feedback

#### Expert task analysis

After the camera read function was validated, the engineers built the first UrApp prototype (Fig. 1). Examining the prototype, the expert panel performed task analysis by first listing all tasks which users must perform after clicking on the UrApp icon (e.g. enter login password, select option for urine test strip read). The panel systematically reviewed each task for potential use issues in a cognitive walk-through (e.g. is the font size too small? Are the instructions are understandable, etc.) and effects on the users if issues occurred. The prototype was then redesigned until the panel agreed that potential use issues had been removed or improved to ensure that they would not impede a user’s ability to operate UrApp or result in negative clinical impact.

#### Simulated use testing

The UrApp prototype was then tested by 10 participants who were either caregivers of patients with nephrotic syndrome < 14 years-old or adolescent patients 14–18 years old. Participants were recruited from the pediatric nephrology clinic at Children’s Healthcare of Atlanta. After consent and assent (where appropriate), users were provided a research iPhone with UrApp already downloaded and asked to perform each panel-identified task (user-app interface functions) and express their opinions of each task by “thinking aloud.” Two research coordinators not involved in the app development observed the users for errors or difficulty performing each task. After the simulated use session, the research coordinators interviewed users for their overall impression of the app, whether they would use the app, and whether they had specific recommendations for improvement. After the interview and testing results, the expert panel met to discuss how UrApp could be redesigned to remove observed use issues and incorporate user suggestions, wherever feasible. After changes were made to each UrApp prototype, another round of user testing and interview was performed with new participants to ensure that all the observed use issues were removed and no additional issues were found.

## Results

### UrApp specifications and functions

**Fig. 3:**
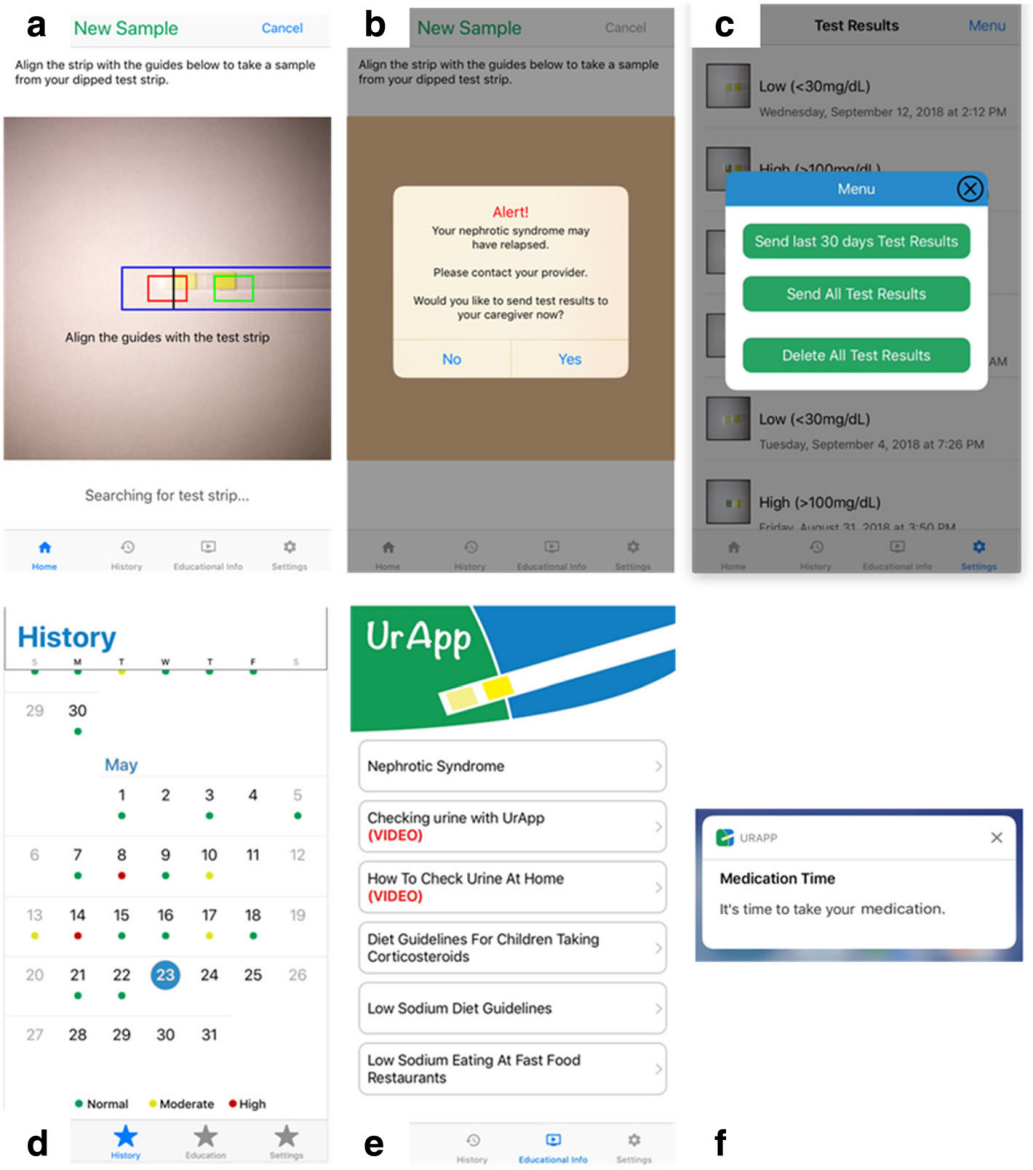
UrApp screenshots displaying key functions: **a** camera read of urine test strips, **b** alerts for significant urine protein findings, **c** transmission of urine protein results, **d** urine protein documentation, **e** educational materials, and **f** medication reminders

**Fig. 4:**
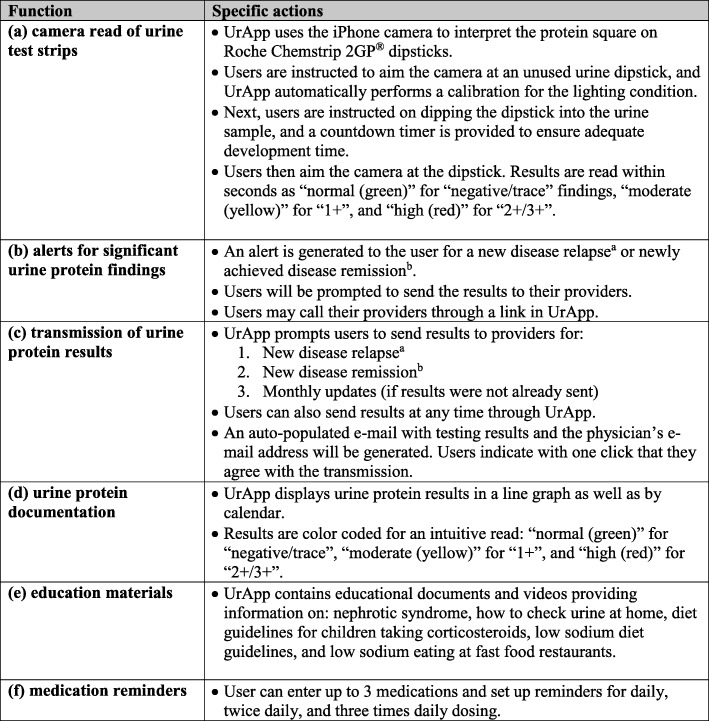
Descriptions of UrApp functions. ROI, region of interest

Figure 3 shows screenshots of UrApp displaying the functions available to users. Figure 4 details the specific actions of each function. UrApp is available for free download on the iPhone App Store (Apple Inc.). All data is stored locally on the phone, without data encryption. Users have to login with a PIN number or by Face/Touch ID each time the app is launched for data security.

### Validity of UrApp urine protein assessment

UrApp camera interpretation of urine protein dipstick results showed excellent agreement with a standard urinalysis machine read (Urisys 1100). Exact agreement occurred in 253/262 (97%) tests using 88 urine samples and three testers (one iPhone 6S and two iPhone 7’s, Table 2). One urine sample with a 2+ (100 mg/dL) protein result by the urinalysis machine was read as “medium” instead of “high” by UrApp in all three phones. Two urine samples with 1+ (30 mg/dL) protein results by the urinalysis machine were read as “normal” instead of “medium” by UrApp in all three phones. The weighted kappa value was 0.91 (95% confidence interval, 0.85–0.97), indicating nearly perfect agreement between UrApp and urinalysis machine read. Agreement between the three phones was 100%. The raw data of all 88 sample protein results by Urisys 1100 and UrApp are presented in Additional file 1: Table S1.

**Table 2:** UrApp camera read of urine protein test strip compared to urinalysis machine read using 88 patient urine samples

Reading Result^a^	Urisys 1100 Total = 88	UrApp (iPhone 7) Total = 88	UrApp (iPhone 7) Total = 88	UrApp (iPhone 6S) Total = 86^b^
Negative/trace (low)	62 (70%)	64 (73%)	64 (73%)	64 (74%)
1+ (medium)	14 (16%)	13 (15%)	13 (15%)	11 (13%)
2+ / 3+ (high)	12 (14%)	12 (14%)	12 (14%)	12 (14%)

^a^Urisys 1100 provides a semi-quantitative read-out of urine protein with levels of: negative, trace, 1+, 2+, and 3+. UrApp was programmed to provide a read-out of “low” for negative and trace readings by standard urinalysis machine read; “medium” for 1+; and “high” for 2+ and 3+ readings

^b^The particular phone was not available for two of the tests

### Usability assessment and user feedback

Two rounds of user testing and interviews were performed with a total of 20 users (15 caregivers and 5 adolescent patients). The UrApp prototype was revised to resolve use issues uncovered during the first round of testing. The second round of testing showed that all 10 users were able to use all prespecified tasks of UrApp without errors. No additional revisions were made to UrApp after the second round of testing.

All 20 participants indicated that they “liked” UrApp. Nineteen of the participants indicated that they thought the app would help them with nephrotic syndrome management and indicated that they would use the app. The one participant who did not think the app would be helpful was a caregiver who had already maintained a computer document for several years containing all urine protein results, medications, and symptoms, and who did not wish to switch to a new system of disease tracking. All participants indicated that they liked the features of UrApp (Figs. 3 and 4). Additional recommendations from the participants that were not incorporated into UrApp due to technical/logistical barriers were: automatic purchase of urine test strips through UrApp; integration of urine test results with electronic medical records; a direct text messaging system to providers within UrApp; a mechanism for multiple users to log in and manage/enter data on separate devices; an Android version of UrApp; and the ability to read other brands of urine test strips.

## Discussion

We described a novel mobile app for childhood nephrotic syndrome monitoring that accurately interprets and tracks urine protein results, facilitates communication between patients and providers, and supports patient education and medication adherence. A key innovative feature of UrApp is the iPhone camera “read” of urine dipstick protein results, which we demonstrated to have high analytic validity. This rapid and accurate interpretation of urine dipsticks ensures that the correct test square is read, removes the need for patients/caregivers to interpret the color change, and removes recall and documentation errors. Our preliminary user testing and feedback results are extremely promising. All participants gave favorable reviews of UrApp, and 19/20 indicated that they found UrApp to be helpful and would use it for nephrotic syndrome management. All 10 users in the final round of testing used UrApp without error and indicated they would be willing to use UrApp.

The use of smartphone-based image processing capabilities to analyze urine test strips has emerged in recent years as an inexpensive and easy-to-use method to support point-of-care testing in the home environment [14–16]. Currently, there is one available FDA-approved urine testing mobile app for the detection of urinary tract infection (Scanwell, Scanwell Health, https://www.scanwellhealth.com/). To our knowledge, UrApp is the first mobile app to utilize the smartphone colorimetric analysis of urine test strips specifically for nephrotic syndrome monitoring, supporting the standard-of-care practice of home urine protein monitoring. UrApp has the potential to improve nephrotic syndrome care by providing patient self-management support and a rapid clinical information system, two crucial componenets of chronic disease care as defined by the Chronic Care Model (MacColl Institute for Healthcare Innovation) [17].

We are aware of one mobile app on the market designed for parents/caregivers of patients with NS that is available for download through Apple App Store and Google Play – Nephrotic Syndrome App by CyberGenie Solutions (http://nephroticsyndromeapp.com/). This app allows users to enter urine protein results and generates a report which can then be sent to providers. UrApp provides urine protein tracking and communication with providers, but also offers reading of urine test strips, educational information, and medication reminders.

Our usability testing is preliminary with a small number of patients. Additional testing is needed to ensure the usability and feasibility of this new tool, as well as to determine UrApp’s ability to improve disease outcomes. Furthermore, our preliminary interview results suggest that users are interested in expanding UrApp functionality including creating an Android version and making it compatible with other urine strip brands. To ensure an effective interventional tool that meets the needs of patients and their caregivers, we are planning a multicentered clinical trial with concurrent process evaluation and stakeholder feedback to systematically address these issues.

## Conclusions

UrApp is a promising tool for nephrotic syndrome management that has the potential to improve disease monitoring and reduce management burden.

## Additional file

Additional file 1: **Table S1.** Raw data of urine test strip protein reads of 88 patient urine samples using a standard urinalysis machine (Urisys 1100) versus UrApp on three iPhones. (DOCX 19 kb)

## Abbreviations

App: Application
ROI: Region of interest
