# Supplementary material for: Development of a novel mobile application to detect urine protein for nephrotic syndrome disease monitoring
Source: BMC Med Inform Decis Mak. 2019 May 30;19:105. doi: 10.1186/s12911-019-0822-z (PMC6543567; doi:10.1186/s12911-019-0822-z)
Supplement: Supplementary file 1 — Table S1. Raw data of urine test strip protein reads of 88 patient urine samples using a standard urinalysis machine (Urisys 1100) versus UrApp on three iPhones. (DOCX 19 kb) [file 12911_2019_822_MOESM1_ESM.docx]

**Additional file 1: Table S1**. Raw data of urine test strip protein reads of 88 patient urine samples using a standard urinalysis machine (Urisys 1100) versus UrApp on three iPhones.

| **Sample** | **Urisys 1100 Read** | **UrApp (iPhone 7) Read** | **UrApp (iPhone 7) Read** | **UrApp (iPhone 6S) Read** |
| --- | --- | --- | --- | --- |
| 1 | negative | normal | normal | normal |
| 2 | 1+ | medium | medium | medium |
| 3 | negative | normal | normal | normal |
| 4 | negative | normal | normal | normal |
| 5 | negative | normal | normal | normal |
| 6 | negative | normal | normal | normal |
| 7 | negative | normal | normal | normal |
| 8 | negative | normal | normal | normal |
| 9 | negative | normal | normal | normal |
| 10 | negative | normal | normal | normal |
| 11 | negative | normal | normal | normal |
| 12 | negative | normal | normal | normal |
| 13 | trace | normal | normal | normal |
| 14 | trace | normal | normal | normal |
| 15 | negative | normal | normal | normal |
| 16 | negative | normal | normal | normal |
| 17 | negative | normal | normal | normal |
| 18 | negative | normal | normal | normal |
| 19 | negative | normal | normal | normal |
| 20 | negative | normal | normal | normal |
| 21 | negative | normal | normal | normal |
| 22 | negative | normal | normal | normal |
| 23 | negative | normal | normal | normal |
| 24 | negative | normal | normal | normal |
| 25 | negative | normal | normal | normal |
| 26 | negative | normal | normal | normal |
| 27 | negative | normal | normal | normal |
| 28 | negative | normal | normal | normal |
| 29 | negative | normal | normal | normal |
| 30 | negative | normal | normal | normal |
| 31 | negative | normal | normal | normal |
| 32 | negative | normal | normal | normal |
| 33 | negative | normal | normal | normal |
| 34 | negative | normal | normal | normal |
| 35 | negative | normal | normal | normal |
| 36 | negative | normal | normal | normal |
| 37 | negative | normal | normal | normal |
| 38 | negative | normal | normal | normal |
| 39 | negative | normal | normal | normal |
| 40 | negative | normal | normal | normal |
| 41 | negative | normal | normal | normal |
| 42 | negative | normal | normal | normal |
| 43 | negative | normal | normal | normal |
| 44 | negative | normal | normal | normal |
| 45 | negative | normal | normal | normal |
| 46 | 2+ | medium | medium | medium |
| 47 | negative | normal | normal | normal |
| 48 | 3+ | high | high | high |
| 49 | negative | normal | normal | normal |
| 50 | 3+ | high | high | high |
| 51 | 3+ | high | high | high |
| 52 | negative | normal | normal | normal |
| 53 | negative | normal | normal | normal |
| 54 | 1+ | medium | medium | * |
| 55 | negative | normal | normal | normal |
| 56 | 1+ | medium | medium | medium |
| 57 | 1+ | medium | medium | * |
| 58 | negative | normal | normal | normal |
| 59 | negative | normal | normal | normal |
| 60 | 1+ | medium | medium | medium |
| 61 | 3+ | high | high | high |
| 62 | negative | normal | normal | normal |
| 63 | trace | normal | normal | normal |
| 64 | negative | normal | normal | normal |
| 65 | negative | normal | normal | normal |
| 66 | 1+ | normal | normal | normal |
| 67 | 1+ | normal | normal | normal |
| 68 | 1+ | medium | medium | medium |
| 69 | 1+ | medium | medium | medium |
| 70 | 1+ | medium | medium | medium |
| 71 | 2+ | high | high | high |
| 72 | 1+ | medium | medium | medium |
| 73 | 2+ | high | high | high |
| 74 | 3+ | high | high | high |
| 75 | 3+ | high | high | high |
| 76 | 3+ | high | high | high |
| 77 | trace | normal | normal | normal |
| 78 | 3+ | high | high | high |
| 79 | 3+ | high | high | high |
| 80 | trace | normal | normal | normal |
| 81 | 1+ | medium | medium | medium |
| 82 | negative | normal | normal | normal |
| 83 | 1+ | medium | medium | medium |
| 84 | 1+ | medium | medium | medium |
| 85 | negative | low | low | low |
| 86 | negative | low | low | low |
| 87 | negative | low | low | low |
| 88 | negative | low | low | low |

Discordant results are highlighted in gray.

*Phone was not available for testing for the specific sample.
